# Predictive Value of QT Interval for Postoperative Atrial Fibrillation in Patients Undergoing Off-Pump Coronary Artery Bypass Surgery

**DOI:** 10.21470/1678-9741-2020-0571

**Published:** 2022

**Authors:** Gencehan Kumtepe, Erhan Renan Ucaroglu

**Affiliations:** 1 Department of Cardiovascular Surgery, Meddem Hospital, Isparta, Turkey; 2 Department of Cardiovascular Surgery, Faculty of Medicine, Abant Izzet Baysal University, Bolu, Turkey

**Keywords:** Atrial Fibrillation, Coronary Artery Bypass, Coronary Artery Bypass, Off-Pump, Elective Surgical Procedures, Postoperative Complications.

## Abstract

**Introduction:**

Postoperative atrial fibrillation (poAF) is a common complication of coronary artery bypass grafting, and its reasons are still the subject of research. The aim of this study was to evaluate whether QT interval is related to new onset of poAF occurrence.

**Methods:**

This study included 167 patients undergoing elective isolated off-pump coronary artery bypass grafting (OPCAB) surgery. Patients were divided into two groups as poAF (+) and poAF (-), according to the development of poAF, and the results of the measurements were compared between the groups.

**Results:**

PoAF was detected in 37 (22.1%) of 167 patients who underwent OPCAB surgery. When QT interval measurements were compared, preoperative and postoperative QT and corrected QT interval (QTc) values were significantly longer in the group with atrial fibrillation. Mean values of preoperative QT were 407.5±27.1 in the poAF (-) group vs. 438.5±48.5 in the poAF (+) group (P<0.001). Mean values of preoperative QTc were 419.1±14.5 in the poAF (-) group vs. 448.5±26.6 in the poAF (+) group (P<0.001). Mean values of postoperative QT were 416.3±48.3 in the poAF (-) group vs. 439.2±45.8 in the poAF (+) group (P=0.005). And mean values of postoperative QTc were 419.8±12.5 in the poAF (-) group vs. 452.0±23.3 in the poAF (+) group (P<0.001).

**Conclusion:**

QT interval measurement may be a new parameter in predicting poAF development after OPCAB surgery.

**Table:** 

Abbreviations, Acronyms & Symbols		
ACE-I = Angiotensin-converting enzyme inhibitors		Hct = Hematocrit
AF = Atrial fibrillation		IABP = Intra-aortic balloon pump
ARB = Angiotensin receptor blockers		ICU = Intensive care unit
AUC = Area under the curve		LA = Left atrial
BMI = Body mass index		LVEF = Left ventricular ejection fraction
CABG = Coronary artery bypass grafting		MPV = Mean platelet volume
CI = Confidence interval		NPV = Negative predictive value
COPD = Chronic obstructive pulmonary disease		OPCAB = Off-pump coronary artery bypass grafting
CRP = C-reactive protein		poAF = Postoperative atrial fibrillation
ECG = Electrocardiogram		PPV = Positive predictive value
FEV1 = Forced expiratory volume in the first second		QTc = Corrected QT interval
FVC = Forced vital capacity		RDW = Red cell distribution width
Hb = Hemoglobin		ROC = Receiver operating characteristic

## INTRODUCTION

Postoperative atrial fibrillation (poAF) is one of the most common complications after cardiac surgery and a major risk factor that can result in mortality of the patients. It complicates about one third of cardiac surgery cases and is associated with major adverse events, prolonged hospitalization, increased healthcare costs, and increased re-hospitalization rates^[1-7]^. The first two weeks after surgery are the most critical, with patients at high risk for poAF^[8]^.

New-onset poAF was shown to predict long-term newly developed atrial fibrillation (AF) in coronary artery bypass grafting (CABG) patients^[9]^. New-onset poAF is significantly associated with increased long-term risk of mortality independent of patient preoperative severity^[10]^.

Although there have been some changes in medical treatment and surgery over time, the overall incidence of poAF has not changed significantly^[11]^. The incidence of poAF remains high, ranging between 15-40%^[12-14]^. The underlying pathophysiology of poAF is still unclear; however, numerous risk factors predisposing to its development have been identified, including advanced age, structural damage to the heart, left ventricular dysfunction, hypertension, and valve surgery^[15]^. Preventive strategies have been partially effective in reducing the overall incidence of AF in the past two decades^[16]^. If the underlying mechanisms can be identified, high-risk patients can be identified, and individual preventive strategies and treatments can be developed^[12]^. Several studies have investigated predictors of poAF^[17,18]^. In a recent meta-analysis including a total of 11 studies and 40,112 patients who underwent CABG, the predictors of poAF were found as older age, low ejection fraction, a history of cardiac failure or hypertension, prior peripheral arterial disease, and stroke^[19]^.

The QT interval is obtained from a standard 12-lead electrocardiogram (ECG) and is a ready, cheap, and fast measure of ventricular repolarization. Several studies have shown that prolonged QT interval is independently correlated with an increased risk of AF and stroke^[20-22]^. In a recent systematic review and meta-analysis, prolonged QT interval was also associated with an increased risk of AF^[19]^. The association between prolongation of QT and AF has been explained by abnormalities in myocardial repolarization^[23]^. However, it is not known whether there is a clinically significant relationship between ventricular repolarization and atrial electrophysiology. Ventricular and atrial refractoriness is determined by many of the same potassium and sodium channels, and there is possibly a correlation between the two^[20]^.

In this study, we aimed to investigate whether a longer QT interval might be an important predictor of poAF in off-pump CABG (OPCAB) patients.

## METHODS

### Study Population and Design

A total of 167 consecutive patients between 18-80 years of age who had no history of AF and underwent elective OPCAB surgery between January 2013 and December 2014 were included in this study. Patients were divided into two groups as poAF (+) and poAF (-), according to the development of poAF, and the results of the measurements were compared between the groups. Demographic data of the patients (age, gender); body mass index (BMI); history of hypertension, chronic kidney failure, diabetes, heart failure, chronic obstructive pulmonary disease (COPD), and stroke; preoperative drug use (angiotensin-converting enzyme inhibitors [ACE-I], angiotensin receptor blockers [ARB], beta blockers, and statins); and echocardiographic findings (ejection fraction, left atrial diameter, and valve disease) were obtained from the hospital records and retrospectively analyzed. All patients were operated by the same surgical team and Octopus IV (Medtronic, Minneapolis) was used as a stabilizer in all operations. Patients who needed preoperative inotropic support, those who underwent on-pump CABG, additional surgical procedures (valves, vascular, etc.), or emergency surgeries were excluded.

### QT Interval and Corrected QT Interval (QTc) Measurements

In each patient, a 12-lead ECG was obtained before the surgery and immediately after extubation, in the postoperative period. QT interval and heart rate were detected on these ECGs and recorded. QTc was measured according to the Bazett formula.

### Postoperative Monitoring

Patient data were retrospectively analysed for the development of new-onset AF in the postoperative period up to discharge. All patients were monitored continuously using five-lead telemetry in the intensive care unit (ICU) postoperatively. Daily routine ECGs were obtained until discharge from the hospital. In addition, when the patients complained of palpitation, dyspnea, and angina, an additional 12-lead ECG was obtained. The new-onset poAF was defined as the new AF requiring treatment during hospitalization after isolated CABG, as defined by the Society of Thoracic Surgeons Adult Cardiac Surgery Database^[11]^.

### Ethical Considerations

The study protocol was approved by the local ethics committee (decision nº: 2020/177, date: 21.07.2020). The study was conducted in accordance with the ethical principles of the Declaration of Helsinki. All patients included in the study were informed and their written consents were taken before the operation.

### Statistical Analysis

Data obtained in this study were statistically analyzed using IBM Corp. Released 2016, IBM SPSS Statistics for Windows, Version 24, Armonk, NY: IBM Corp statistical software. To calculate the sample width, power of analysis for each variable was determined by taking at least 0.80 and Type 1 error 0.05. Descriptive statistics for continuous variables are expressed as median, mean, standard deviation, minimum, and maximum; and descriptive statistics for categorical variables, as numbers and percentages.

Kolmogorov-Smirnov and Shapiro-Wilk tests were used to check whether the measurement averages were normally distributed and nonparametric tests were applied since the measurement values of the variables were not normally distributed. Mann-Whitney U Test was used to compare the measurements of the AF groups. Wilcoxon test was used for nonparametric variables in comparisons of dependent variables.

In order to determine predictive values of measurements according to AF groups, the area under the curve (AUC), sensitivity-specificity values, and cutoff values were determined by receiver operating characteristic (ROC) analysis. Chi-square test was used to determine the relationship between categorical variables. P<0.05 values were considered statistically significant.

## RESULTS

The present study included 167 consecutive patients who had no prior history of AF and underwent OPCAB. Thirty-seven of 167 patients (22.1%) developed AF before the discharge from the hospital in the postoperative period. Mean age was 66.7±9.0 years in the poAF (+) group and 61.7±10.4 years in the poAF (-) group. The mean age was statistically significantly higher in the poAF (+) group compared to poAF (-) group (P=0.007). Of the 37 patients who developed poAF, nine (24.3%) were female and 28 (75.7%) were male. Of the 130 patients without poAF, 24 (18.5%) were female and 106 (%81.5) were male. No significant difference was found between both groups in terms of gender distribution (P>0.05). In addition, there was no significant difference between the groups in terms of BMI (P>0.05). And no statistically significant difference was found between both groups in terms of frequency of comorbidities (diabetes mellitus, hypertension, hyperlipidemia, COPD, myocardial infarction, and smoking status) and drug use (beta blocker therapy, statin therapy, ACE-I/ARB therapy) (for all P>0.05) (Table 1).

**Table 1:** Preoperative clinical characteristics of the groups.

		poAF (-) group	poAF (+) group	*P*-value
		n=130	n=37	
Age (years)		61.7±10.4	66.7±9.0	0.007^*^
BMI		27.7±5.0	28.6±4.7	0.331^*^
Gender, n (%)	Female	24 (18.5)	9 (24.3)	0.429^#^
	Male	106 (81.5)	28 (75.7)	
Diabetes mellitus, n (%)	-	73 (56.2)	16 (43.2)	0.165^#^
	+	57 (43.8)	21 (56.8)	
Hypertension, n (%)	-	69 (53.1)	16 (43.2)	0.291^#^
	+	61 (46.9)	21 (56.8)	
Smoking, n (%)	-	67 (51.5)	19 (51.4)	0.984^#^
	+	63 (48.5)	18 (48.6)	
Hyperlipidemia, n (%)	-	46 (35.4)	15 (40.5)	0.566^#^
	+	84 (64.6)	22 (59.5)	
COPD, n (%)	-	123 (94.6)	34 (91.9)	0.538^#^
	+	7 (5.4)	3 (8.1)	
Myocardial infarction, n (%)	-	92 (70.8)	21 (56.8)	0.108^#^
	+	38 (29.2)	16 (43.2)	
Beta blocker therapy, n (%)	-	120 (92.4)	32 (86.5)	0.428^#^
	+	10 (7.6)	5 (13.5)	
Statin therapy, n (%)	-	42 (32.4)	9 (24.3)	0.527^#^
	+	88 (67.6)	28 (75.7)	
ACE-I/ARB therapy, n (%)	-	57 (43.9)	15 (40.6)	0.608^#^
	+	73 (56.1)	22 (59.4)	

* Significance levels according to Mann-Whitney U test results.

# Significance levels according to the Chi-square test results.

ACE-I=angiotensin-converting enzyme inhibitors; ARB=angiotensin receptor blockers; BMI=body mass index; COPD=chronic obstructive pulmonary disease; poAF=postoperative atrial fibrillation

When pre- and postoperative laboratory data and clinical findings were evaluated, no statistically significant difference was observed between both groups in terms of preoperative left ventricular ejection fraction, preoperative left atrial diameter, preoperative hemoglobin (Hb), preoperative hematocrit (Hct), preoperative red cell distribution width (RDW), preoperative mean platelet volume (MPV), preoperative C-reactive protein, preoperative troponin I, postoperative Hb, postoperative Hct, and postoperative troponin I (for all P>0.05).

**Fig 1:**
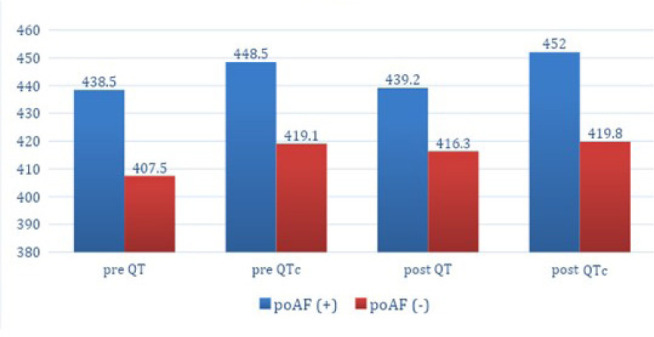
Pre- and postoperative QT and corrected QT interval (QTc) values of postoperative atrial fibrillation (poAF) (+) and poAF (-) groups.

On the contrary, preoperative QT (P<0.001), preoperative QTc (P<0.001), postoperative QT (P=0.005), and postoperative QTc values were statistically significantly higher in the poAF (+) group compared to the poAF (-) group (Table 2, Figure 1).

**Table 2:** Laboratory and other parameters of the groups.

	poAF (-) group	poAF (+) group	^*^P-value
	n=130	n=37	
Preoperative LVEF	51.8±9.0	48.7±9.1	0.053
Preoperative left atrial diameter	38.2±5.7	40.1±6.0	0.083
Preoperative QT	407.5±27.1	438.5±45.8	< 0.001
Preoperative QTc	419.1±14.5	448.5±26.6	< 0.001
Postoperative QT	416.3±48.3	439.2±45.8	0.005
Postoperative QTc	419.8±12.5	452.0±23.3	< 0.001
Preoperative Hb	13.4±2.0	13.7±2.1	0.577
Preoperative Hct	40,8±5,9	41,3±6,0	0.906
Preoperative RDW	16.3±2.2	16.4±2.7	0.857
Preoperative MPV	8.2±1.6	8.2±1.4	0.740
Preoperative CRP	18.0±29.6	13.0±27.4	0.140
Preoperative troponin I	980.3±3095.1	350.6±849.0	0.900
Postoperative Hb	10.6±1.3	10.9±1.4	0.389
Postoperative Hct	31.6±3.7	32.5±4.7	0.480
Postoperative troponin I	6961.6±13958.9	8311.6±14162.2	0.197

* Significance levels according to Mann-Whitney U test results.

CRP=C-reactive protein; Hb=hemoglobin; Hct=hematocrit; LVEF=left ventricular ejection fraction; MPV=mean platelet volume; poAF=postoperative atrial fibrillation; QTc=corrected QT interval; RDW=red cell distribution width

When operative and postoperative characteristics of CABG operations were evaluated, no statistically significant difference was found between poAF (+) and poAF (-) groups in terms of heart rate, forced expiratory volume in the first second/forced vital capacity, bypassed vessels, transfusion (unit), length of stay in ICU (hours), hospital stay (days), extubation time (hours), postoperative lactate, the need for transfusion, the presence of intra-aortic balloon pump, the need for inotropic support, neurologic deficit, and mortality (for all P>0.05). On the other hand, the amount of total drainage was statistically significantly higher in the poAF (+) group than in the poAF (-) group (P=0.034) (Table 3).

**Table 3:** Operative and postoperative data of the groups.

		poAF (-) group	poAF (+) group	*P*-value
		n=130	n=37	
Heart rate		64.2±6.9	64.3±9.4	0.869^*^
FEV1/FVC		77.1±10.6	74.9±13.3	0.344^*^
Bypassed vessel		4.6±1.0	4.5±1.1	0.386^*^
Transfusion (unit)		2.0±1.4	1.7±1.0	0.444^*^
Total drainage		579.1±298.1	675.8±279.4	0.034^*^
Intensive care unit (hours)		69.6±45.0	71.4±42.3	0.623^*^
Hospital stay (days)		3.3±2.2	3.0±2.0	0.635^*^
Extubation time (hours)		6.3±3.0	5.9±1.7	0.678^*^
Postoperative lactate		1.5±0.7	2.3±3.1	0.508^*^
Transfusion	-	72 (56.3)	22 (59.5)	0.728^#^
	+	56 (43.8)	15 (40.5)	
IABP	-	123 (94.6)	35 (94.6)	0.996^#^
	+	7 (5.4)	2 (5.4)	
Inotropic support	-	109 (83.8)	27 (73.0)	0.133^#^
	+	21 (16.2)	10 (27.0)	
Neurologic deficit	-	127 (97.7)	36 (97.3)	0.890^#^
	+	3 (2.3)	1 (2.7)	
Mortality	+	2 (1.5)	1 (2.7)	0.638^#^
	-	128 (98.5)	36 (97.3)	

* Significance levels according to Mann-Whitney U test results.

# Significance levels according to the Chi-square test results.

FEV1=forced expiratory volume in the first second; FVC=forced vital capacity; IABP=intra-aortic balloon pump; poAF=postoperative atrial fibrillation

Risk factors for the development of poAF were evaluated by univariate logistic regression analysis. Age (odds ratio [95% confidence interval, CI] 1.062 [1.015-1.111], P=0.009) and postoperative QTc (odds ratio [95% CI] 1.147 [1.064-1.238], P=0.001) were detected to be significantly higher for poAF. In multivariate logistic regression analysis, only postoperative QTc was found to be an independent predictor for the development of poAF (odds ratio [95% CI] 1.093 [1.063-1.122], P=0.001) (Table 4).

**Table 4:** Univariate and multivariate logistic regression analyses of independent parameters for atrial fibrillation.

	Univariate		Multivariate	
Variables	Odds ratio (95% CI)	*P*-value	Odds ratio (95% CI)	*P*-value
Age	1.062 (1.015-1.111)	0.009	1.013 (0.961-1.069)	0.621
Postoperative QTc	1.147 (1.064-1.238)	0.001	1.093 (1.063-1.122)	0.001
Preoperative QTc	0.973 (0.905-1.045)	0.448		
Postoperative QT	0.994 (0.980-1.009)	0.438		
Preoperative QT	0.992 (0.968-1.017)	0.540		
LVEF	0.979 (0.921-1.041)	0.500		
LA diameter	1.095 (0.997-1.203)	0.058		
Total drainage	0.999 (0.997-1.002)	0.564		

CI=confidence interval; LA=left atrial; LVEF=left ventricular ejection fraction; QTc=corrected QT interval

**Fig 2:**
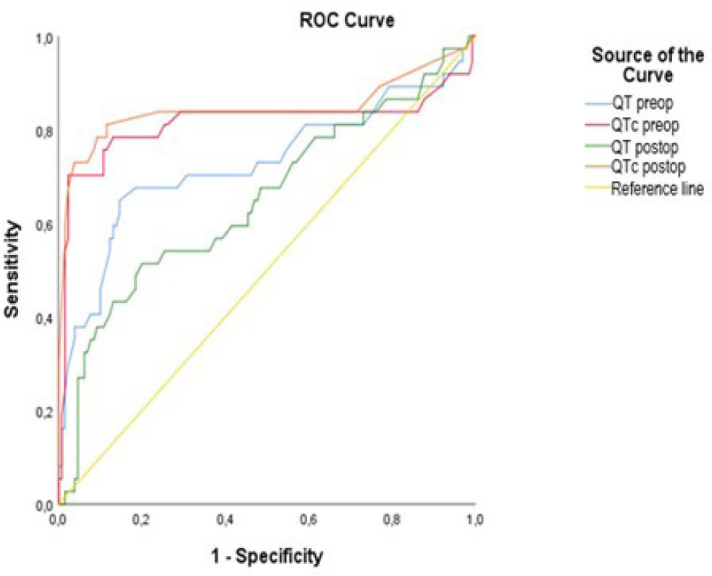
Receiver operating characteristic (ROC) curve and the area under the curve for QT and corrected QT interval (QTc) to predict postoperative atrial fibrillation.

In the ROC curve analysis we performed to determine cutoff values, sensitivity and specificity values of QT and QTc, and AUC cutoff, sensitivity and specificity values were calculated for preoperative QT, preoperative QTc, postoperative QT, and postoperative QTc in predicting poAF at 95% CI and are shown in Table 5 and Figure 2. In addition, after ROC analysis of four QT values was performed, a pairwise analysis was also performed for the ROC curves. The results of pairwise analysis are shown in Table 6 and the result of cutoff values, positive predictive value, and negative predictive value are shown in Table 7.

**Table 5:** Area under the curve (AUC), cutoff value, and diagnostic tests according to atrial fibrillation groups.

Test results	AUC	Standard error	*P*-value	Cutoff	Sensitivity	Specificity	95% Confidence interval	
Variables							Lower	Upper
Preoperative QT	0.728	0.057	0.001	419	0.703	0.692	0.617	0.839
Preoperative QTc	0.814	0.057	0.001	434.5	0.784	0.869	0.703	0.925
Postoperative QT	0.651	0.056	0.005	423.5	0.595	0.585	0.541	0.76
Postoperative QTc	0.845	0.051	0.001	431	0.811	0.885	0.744	0.945

QTc=corrected QT interval

**Table 6:** Paired comparison results of variables with significant cutoff values after ROC analysis.

		N	Mean	Standard deviation	Min.	Max.	^*^P-value
Preoperative QT	AF (+)	130	407,4923	27,11281	290	510	< 0,001
	AF (-)	37	438,5135	45,84431	348	538	
	Total	167	414,3653	34,55454	290	538	
Preoperative QTc	AF (+)	130	419,1154	14,52271	374	474	< 0,001
	AF (-)	37	448,4865	26,62666	389	491	
	Total	167	425,6228	21,61788	374	491	
Postoperative QT	AF (+)	130	416,3	48,32363	168	592	0,011
	AF (-)	37	439,2162	45,82463	322	537	
	Total	167	421,3772	48,59218	168	592	
Postoperative QTc	AF (+)	130	419,8	12,48093	380	462	<0,001
	AF (-)	37	452	23,29998	401	486	
	Total	167	426,9341	20,46188	380	486	

* Significance levels according to independent *t*-test results.

AF=atrial fibrillation; QTc=corrected QT interval; ROC=receiver operating characteristic

**Table 7:** Cutoff levels for QT and QTc preoperative and postoperative measurements in atrial fibrillation groups.

	Cutoff	Sensitivity % (95% CI)	Sensitivity % (95% CI)	PPV %	NPV %	AUC	*P*-value
Preoperative QT	419,0	70,3 (59,4-79,3)	69,2 (41,3-87,8)	39,4	89,1	0,728	0,001
Preoperative QTc	434,5	78,4 (68,5-86,1)	86,9 (59,1-96,8)	65,1	9,34	0,814	0,001
Postoperative QT	423,5	59,5 (48,4-69,7)	58,5 (32,1-80,8)	29	83,5	0,651	0,005
Postoperative QTc	431,0	81,1 (71,0-88,3)	88,5 (60,9-97,4)	66,7	94,3	0,845	0,001

AUC=area under the curve; CI=confidence interval; NPV=negative predictive value; PPV=positive predictive value; QTc=corrected QT interval

## DISCUSSION

In the present study, preoperative QT, preoperative QTc, postoperative QT, and postoperative QTc interval measurements were significantly longer in patients who underwent OPCAB surgery and developed AF in the postoperative period. In addition, advanced age was found to be an independent risk factor for the development of poAF. In a study by Velioglu et al.^[24]^ investigating the development of poAF after beating heart CABG, age was significantly higher in the poAF (+) group. In a study by Turkkan and Bozbeyoğlu, conducted on 311 patients undergoing elective CABG, poAF (+) patients were significantly older than poAF (-) patients^[25]^. In another study, the rate of patients aged > 85 years was significantly higher in the poAF (+) group^[26]^. And in a study by Lotfi et al.^[27]^, the mean age was statistically significantly higher in poAF (+) patients. As is seen, older age is a risk factor of developing poAF in most of the studies. In addition, age is the only risk factor that has been systematically proven in the literature^[28]^.

No statistically significant difference was found between poAF (+) and poAF (-) groups in terms of comorbidities (diabetes mellitus, hypertension, hyperlipidemia, COPD, myocardial infarction, and smoking status) in the present study. Conversely, there are studies in the literature that have reported several comorbidities as risk factors for poAF including a history of prior AF, hypertension, congestive heart failure, COPD, chronic lung disease, and chronic kidney disease^[17,29]^. However, many of these studies are retrospectively designed and have a relatively short follow-up. Findings of the studies regarding other possible preoperative risk factors are inconsistent^[30-32]^. Therefore, we still do not have an accurate understanding about the association between comorbidities and poAF. For example, unlike the other studies, Akintoye et al.^[33]^ found that the frequency of hypertension and prior history of AF were significantly higher in poAF (-) patients.

In this study, no correlation was found between preoperative RDW and preoperative MPV values and poAF development. In another previous study, it was found that preoperative RDW and preoperative MPV levels did not have a predictive value for poAF in patients undergoing OPCAB surgery^[34]^.

Studies have investigated routine ECG parameters as potential predictors of incident AF. Perez et al.^[35]^ identified several *P*-wave characteristics - including *P*-wave index - that independently increased the risk of incident AF.

The prolonged QT interval, previously thought to be associated only with ventricular arrhythmias, has been associated with an increased risk of AF incidence^[20]^. In a study conducted to determine the characteristics of cardiac autonomic modulation and repolarization, preoperative QT and QTc intervals were found to be longer in the group that developed poAF^[36]^.

Nielsen et al.^[21]^ investigated the development of AF in subjects who were followed for an average of 5.7 years in a study in the general population. Compared to the reference group (40th to < 60th percentile, 411 to 419 ms), in the 99th percentile and over (≥ 464 ms) and 1st percentile and below (≤ 372 ms) groups, there was a statistically significant increased risk of AF in QTcFram (QTc calculated using the Framingham formula) intervals. When lone AF subgroup analysis was performed, it was revealed that the relationship between the QTc interval and the lone AF result was at least stronger for the QTc intervals in the upper range compared to AF.

Patel et al.^[37]^ compared the QT interval components (QRS duration and JT interval) with the incidence of AF. In the study in which 4,181 participants were analyzed, it was found that the JT interval is a more important marker of AF risk in the QT interval among other personal delays^[38]^. In another study with 14,625 participants, 1,505 (10.3%) developed AF in mean 17.6 years of follow-up. When the ECG parameters of patients who developed AF were examined, QT-interval components involved

### Limitations

Retrospective study design and lack of follow-up after discharge are important limitations of the study. In addition, patients who developed short-term AF attacks may not be detected because there was no continuous ECG monitoring. Since it is performed without excluding some risk factors for the development of AF, we cannot clearly express the relationship between the QT interval and poAF. Further studies are needed to support our findings.

## CONCLUSION

In this study, a strong relationship was detected between QT interval prolongation and poAF. Qt interval measurement is a simple and cost-free process. It is clear that there is a need for a prospective, randomized studies, with and larger number of patients to prove the relationship between the QT interval and its components and poAF. If this relationship can be detected, more effective preventive and therapeutic strategies can be developed as patients at risk for the development of poAF can be identified in advance.

in repolarization, but not depolarization, exhibited significant associations with incident AF. Kinoshita et al.^[39]^ determined that preoperative QT interval is an independent predictor of overall death and sudden cardiac death after coronary bypass surgery.

In the present study, we found that preoperative QT (cutoff: 419, sensitivity: 0.703, specificity: 0.692), preoperative QTc (cutoff: 434.5, sensitivity: 0.784, specificity: 0.869), postoperative QT (cutoff: 423.5, sensitivity: 0.595, specificity: 0.585), and postoperative QTc (cutoff: 419, sensitivity: 0.703, specificity: 0.692) were potential predictors of poAF. However, our results should be supported by larger series prospective studies.

## References

[1] Hogue CW Jr, Creswell LL, Gutterman DD, Fleisher LA (2005). American College of Chest Physicians. Epidemiology, mechanisms,
and risks: American college of chest physicians guidelines for the
prevention and management of postoperative atrial fibrillation after cardiac
surgery. Chest.

[2] Mitchell LB; CCS Atrial Fibrillation Guidelines Committee (2011). Canadian cardiovascular society atrial fibrillation guidelines
2010: prevention and treatment of atrial fibrillation following cardiac
surgery. Can J Cardiol.

[3] Lahey SJ, Campos CT, Jennings B, Pawlow P, Stokes T, Levitsky S (1998). Hospital readmission after cardiac surgery. Does "fast track" cardiac surgery result in cost saving or cost
shifting? Circulation.

[4] Aksoy F, Uysal D, Ibrişim E (2020). Relationship between c-reactive protein/albumin ratio and
new-onset atrial fibrillation after coronary artery bypass
grafting. Rev Assoc Med Bras (1992).

[5] Ronsoni RM, Souza AZM, Leiria TLL, Lima GG (2020). Update on management of postoperative atrial fibrillation after
cardiac surgery. Braz J Cardiovasc Surg.

[6] Cerit L, Özcem B, Cerit Z, Duygu H (2018). Preventive effect of preoperative vitamin D supplementation on
postoperative atrial fibrillation. Braz J Cardiovasc Surg.

[7] Aksoy F, Uysal D, Ibrişim E (2020). Predictive values of C-reactive protein/albumin ratio in
new-onset atrial fibrillation after coronary artery bypass
grafting. Rev Assoc Med Bras (1992).

[8] Agrawal DK, Boosani CS (2017). Gene therapy to keep the QT rhythms "on the QT". J Thorac Cardiovasc Surg.

[9] Lee SH, Kang DR, Uhm JS, Shim J, Sung JH, Kim JY (2014). New-onset atrial fibrillation predicts long-term newly developed
atrial fibrillation after coronary artery bypass graft. Am Heart J.

[10] Filardo G, Hamilton C, Hebeler RF Jr (2009). Hamman B, Grayburn P. New-onset postoperative atrial fibrillation
after isolated coronary artery bypass graft surgery and long-term
survival. Circ Cardiovasc Qual Outcomes.

[11] Turagam MK, Mirza M, Werner PH, Sra J, Kress DC, Tajik AJ (2016). Circulating biomarkers predictive of postoperative atrial
fibrillation. Cardiol Rev.

[12] Elahi M, Hadjinikolaou L, Galiñanes M (2003). Incidence and clinical consequences of atrial fibrillation within
1 year of first-time isolated coronary bypass surgery. Circulation.

[13] Villareal RP, Hariharan R, Liu BC, Kar B, Lee VV, Elayda M (2004). Postoperative atrial fibrillation and mortality after coronary
artery bypass surgery. J Am Coll Cardiol.

[14] Yüksel A, Velioglu Y, Tecimer ME, Kan II, Bicer M, Gurbuz O (2019). Is there any relationship of postoperative atrial fibrillation
with the use of blood products and postoperative hemoglobin levels in
patients undergoing coronary artery bypass grafting?. Medicine Science.

[15] Yadava M, Hughey AB, Crawford TC (2016). Postoperative atrial fibrillation: incidence, mechanisms, and
clinical correlates. Heart Fail Clin.

[16] Shen J, Lall S, Zheng V, Buckley P, Damiano RJ Jr, Schuessler RB (2011). The persistent problem of new-onset postoperative atrial
fibrillation: a single-institution experience over two
decades. J Thorac Cardiovasc Surg.

[17] Mathew JP, Fontes ML, Tudor IC, Ramsay J, Duke P, Mazer CD (2004). A multicenter risk index for atrial fibrillation after cardiac
surgery. JAMA.

[18] Zaman AG, Archbold RA, Helft G, Paul EA, Curzen NP, Mills PG (2000). Atrial fibrillation after coronary artery bypass surgery: a model
for preoperative risk stratification. Circulation.

[19] Kaw R, Hernandez AV, Masood I, Gillinov AM, Saliba W, Blackstone EH (2011). Short- and long-term mortality associated with new-onset atrial
fibrillation after coronary artery bypass grafting: a systematic review and
meta-analysis. J Thorac Cardiovasc Surg.

[20] Mandyam MC, Soliman EZ, Alonso A, Dewland TA, Heckbert SR, Vittinghoff E (2013). The QT interval and risk of incident atrial
fibrillation. Heart Rhythm.

[21] Nielsen JB, Graff C, Pietersen A, Lind B, Struijk JJ, Olesen MS (2013). J-shaped association between QTc interval duration and the risk
of atrial fibrillation: results from the Copenhagen ECG
study. J Am Coll Cardiol.

[22] Soliman EZ, Howard G, Cushman M, Kissela B, Kleindorfer D, Le A (2012). Prolongation of QTc and risk of stroke: The REGARDS (REasons for
Geographic and Racial Differences in Stroke) study. J Am Coll Cardiol.

[23] Johnson JN, Tester DJ, Perry J, Salisbury BA, Reed CR, Ackerman MJ (2008). Prevalence of early-onset atrial fibrillation in congenital long
QT syndrome. Heart Rhythm.

[24] Velioglu Y, Yuksel A (2019). Predictors of postoperative atrial fibrillation after
beating-heart coronary artery bypass surgery: is cardiopulmonary bypass a
risk factor?. Acta Cardiol Sin.

[25] Turkkan C and Bozbeyoglu E (2018). Impact of Electrocardiographic Diastolic Parameters and Diastolic
ECG Index in Predicting Postoperative Atrial Fibrillation. Int J Basic Clin Studies.

[26] Sigurdsson MI, Longford NT, Heydarpour M, Saddic L, Chang TW, Fox AA (2016). Duration of postoperative atrial fibrillation after cardiac
surgery is associated with worsened long-term survival. Ann Thorac Surg.

[27] Lotfi A, Wartak S, Sethi P, Garb J, Giugliano GR (2011). Postoperative atrial fibrillation is not associated with an
increase risk of stroke or the type and number of grafts: a single-center
retrospective analysis. Clin Cardiol.

[28] Perrier S, Meyer N, Hoang Minh T, Announe T, Bentz J, Billaud P (2017). Predictors of atrial fibrillation after coronary artery bypass
grafting: a bayesian analysis. Ann Thorac Surg.

[29] Sun X, Boyce SW, Hill PC, Bafi AS, Xue Z, Lindsay J (2011). Association of body mass index with new-onset atrial fibrillation
after coronary artery bypass grafting operations. Ann Thorac Surg.

[30] Magee MJ, Herbert MA, Dewey TM, Edgerton JR, Ryan WH, Prince S (2007). Atrial fibrillation after coronary artery bypass grafting
surgery: development of a predictive risk algorithm. Ann Thorac Surg.

[31] Hernandez AV, Kaw R, Pasupuleti V, Bina P, Ioannidis JP, Bueno H (2013). Association between obesity and postoperative atrial fibrillation
in patients undergoing cardiac operations: a systematic review and
meta-analysis. Ann Thorac Surg.

[32] Tayyareci Y, Yildirimtürk O, Aytekin V, Memic K, Behramoglu F, Demiroglu IC (2010). Preoperative left atrial mechanical dysfunction predicts
postoperative atrial fibrillation after coronary artery bypass graft
operation - a velocity vector imaging-based study -. Circ J.

[33] Akintoye E, Sellke F, Marchioli R, Tavazzi L, Mozaffarian D (2018). Factors associated with postoperative atrial fibrillation and
other adverse events after cardiac surgery. J Thorac Cardiovasc Surg.

[34] Ozsin KK, Sanri US, Toktas F, Yavuz S. (2018). Relationship between red cell distribution width and mean
platelet volume with new onset atrial fibrillation afteroff-pump coronary
artery bypass grafting. Bratisl Lek Listy.

[35] Perez MV, Dewey FE, Marcus R, Ashley EA, Al-Ahmad AA, Wang PJ (2009). Electrocardiographic predictors of atrial
fibrillation. Am Heart J.

[36] Kališnik JM, Hrovat E, Hrastovec A, Avbelj V, Žibert J, Geršak B (2015). Severe cardiac autonomic derangement and altered ventricular
repolarization pave the way to postoperative atrial
fibrillation. Innovations (Phila).

[37] Patel N, O'Neal WT, Whalen SP, Soliman EZ (2018). The association of QT interval components with atrial
fibrillation. Ann Noninvasive Electrocardiol.

[38] Roberts JD, Soliman EZ, Alonso A, Vittinghoff E, Chen LY, Loehr L (2017). Electrocardiographic intervals associated with incident atrial
fibrillation: dissecting the QT interval. Heart Rhythm.

[39] Kinoshita T, Asai T, Suzuki T, Matsubayashi K, Horie M (2012). Time course and prognostic implications of QT interval in
patients with coronary artery disease undergoing coronary bypass
surgery. J Cardiovasc Electrophysiol.

